# Improving the approach to assess impact of anaemia control programs during pregnancy in India: a critical analysis

**DOI:** 10.1186/s12884-022-05248-z

**Published:** 2022-12-26

**Authors:** Sutapa Bandyopadhyay Neogi, Ameet Babre, Mini Varghese, Jennifer Busch Hallen

**Affiliations:** 1 International Institute of Health Management Research (IIHMR Delhi), Delhi, India; 2 Nutrition International, India office, New Delhi, India; 3 Nutrition International, Ottawa, Canada

**Keywords:** Anaemia, Prevalence, Pregnancy, Developing countries, Health systems, Health programs

## Abstract

Around 42.7% of women experience anaemia during pregnancy in low- and middle-income countries. Countries in southeast Asia (with prevalence ranging between 40 and 60%) have reported a modest decline over the past 25 years. Nearly half the pregnant women continue to be anaemic in India between 2005-06 and 2015-16, although severe anaemia has reduced from 2.2% to 1.3%.India has been committed to achieving a target of 32% prevalence of anaemia in pregnant women from 50% by 2022. There are concerns around stagnancy in the prevalence of anaemia in pregnancy despite a strong political commitment. The paper puts forth the arguments that should be considered while introspecting why India might run the risk of not achieving the expected reduction. The reported findings highlight several methodological issues such as hemoglobin cut-offs used to determine anaemia during pregnancy, method of estimation of Hb, and less emphasis on causes other than iron deficiency anemia.

## Background

Anaemia continues to be a public health problem affecting the health of mothers and newborns across the world. Around 42.7% (95% CI: 37.0, 48.4%) of women have anaemia during pregnancy in low- and middle-income countries [1]. The World Health Organization Global Nutrition Targets call for a 50% reduction in anaemia in women of reproductive age by 2025 relative to 2011 prevalence [2].

India has been committed to achieving a target of 32% prevalence of anaemia in pregnant women by 2022 [from 50% baseline as per National Family Health Survey (NFHS) 4 data] and 40% prevalence in lactating non pregnant women (from 58% prevalence as per NFHS 4) [3]. Data from NFHS 5 (2019–20) survey do not show any appreciable impact on the prevalence of anaemia in pregnancy.

Program implementers and policy makers are concerned around stagnancy in the prevalence of anaemia in pregnancy despite political commitment and implementation of national anaemia control program for decades. The objective of the paper is to put forth the arguments that should be considered while introspecting on our approach to assess impact of anemia reduction efforts.

## Approach and analysis

The analysis is based on the secondary review of literature (reference period 2010 till 2021). The trends in the prevalence of anaemia were analyzed from nation-wide NFHS surveys. These are large-scale, multi-stage, multi-round surveys conducted periodically in a representative sample of households throughout India to provide national, state, district level estimates. Results were interpreted in the light of prevalence reported across different states, methods of measurement of Hemoglobin (Hb), and case definitions used in the reports. For understanding the causes of anemia, its determinants and consequences, published systematic reviews, meta-analysis and observational studies were considered. The findings were compiled and interpreted to understand the modifiable/ non-modifiable, nutritional/ non nutritional factors. A review of global policies was done to understand the guidelines on anemia prevention and management guidelines. A comparative analysis with national guidelines was done to explore the similarities and differences, especially with regards to the cut-offs used.

The Government of India has been addressing the problem of anemia through its national program since the past few decades. The National Nutritional Anaemia Prophylaxis Programme (NNAPP) was launched in 1973 that aimed at iron and folic acid supplementation during pregnancy. Since 1991, the National Anaemia Control Programme (NACP) emphasized diagnosis and management of anaemia across all health care settings in the entire country. Tenth Five Year Plan highlighted universal screening of pregnant women for anaemia followed by providing appropriate management. National Iron Plus Initiative (NIPI) elaborated how the programme should be implemented [4]. This was followed by the launch of Anaemia Mukt Bharat (AMB) to provide boost to the program. Despite all these efforts, both supply and demand side issues like poor uptake of antenatal care (ANC) facilities by pregnant women, failure to screen pregnant women for anaemia, poor compliance regarding consumption of IFA [5] led to several concerns around implementation of the program.

Reduction of anaemia is one of the important objectives of the POSHAN (Prime Minister’s Overarching Scheme for Holistic Nutrition) Abhiyaan launched in March 2018. Complying with the targets of POSHAN Abhiyaan and National Nutrition Strategy set by National Institution for Transforming India (NITI) Aayog, the AMB strategy has been designed to reduce prevalence of anaemia by 3 percentage points per year among children, adolescents, and women in the reproductive age group (15–49 years), between the year 2018 and 2022.

We present here methodological issues worth considering before providing any conclusive evidence on the success or failure of any program.

1. **Findings of survey methods review**: Survey design, sampling procedure and sample size as well as margin of error around estimates assumed in different surveys can have strong bearing on the estimates of prevalence of any parameter. District Level Health Survey (DLHS) and Annual Health Survey (AHS) in India are well designed large-scale surveys comparable to NFHS in terms of methodology and robustness. Despite these similarities, the variations in prevalence of anaemia in pregnancy both at the national and State level between NFHS 2, 3 and 4 on one side and DLHS 2, 4 and AHS on the other, were beyond 25% and were unlikely to be due to differences in the survey methodology [4]. For example, the prevalence of anemia in NFHS 2 was 49.7 compared to 94.8 as reported in DLHS 2.
  One reason for the difference is the criteria used for grading anaemia. The NHFS 2, 3 and 4 graded anaemia according to the WHO grading of anaemia (Hb ≥11 g/dl: no anaemia, 10.0–10.9 g/dl- mild anaemia, 7.0–9.9 g/dl:moderate anaemia, < 7.0 g/dl:severe anaemia). DLHS used the grading of anaemia based on functional decompensation which has been associated with a fall in hemoglobin (Hb) levels (Hb ≥11 g/dl: no anaemia; 8.0–10.9 g/dl: mild anaemia, 5.0–7.9 g/dl: moderate anaemia, < 5.0 g/dl: severe anaemia) [4]. Different surveys provide an opportunity to triangulate results but differing case definitions in this case preclude it.
  Anaemia is diagnosed objectively using HemoCue on a predetermined sample of population by measuring Hb in NFHS surveys. However it is reported that due to different models of HemoCue analyzers being used for NFHS-2,3 and 4 consistent results of Hb estimations were not produced by the earlier models of the machine [6]. Apart from that, inaccuracies could result from use of capillary blood as venous blood would provide better estimates.
  Another explanation for the differences between NFHS and DLHS surveys was because DLHS utilized cyanmethaemoglobin method [6, 7]. Precision of HemoCue readings have been questioned by NFHS reports [8]. Recent evidence indicates that it overestimates Hb by 0.70 g/dl (0.58 to 0.82) in moderate and severe anaemia combined and by 0.84 g/dl (0.66 to 1.03) in cases of severe anaemia [9].
  Based on these findings it may seem that although NFHS surveys reflect the relative trend of the prevalence, it may not be accurate in ascertaining the prevalence of anaemia, severe anaemia (Hb < 7 g%) in particular. Among women of reproductive age group, severe anaemia showed an increase in NFHS 3 compared to NFHS 2 but then showed a decline in NFHS 4 (Table 1). The prevalence of severe anaemia in pregnancy has reduced considerably between NFHS 3 and 4 (Table 2). Whether the decline is true or spurious remains a matter of concern owing to measurement errors. Severe anaemia diagnosis and management are the mainstay for reducing mortality due to anaemia [10].
2. **Measures of impact of anaemia control programs**:
  Management of anaemia in pregnancy cannot reduce anaemia in pregnancy because of the time required to raise Hb levels. Reduction in prevalence of anaemia in pregnancy can be best achieved by targeting women during the pre-pregnancy period.
  Iron supplementation during pregnancy is rather a measure to treat anaemia and prevent adverse consequences resulting from anaemia (mortality and morbidity due to anaemia including haemorrhage). NNACP strongly focuses on treatment of anaemia during pregnancy. An indirect way of impact assessment could be anaemia related maternal mortality. In the absence of any reliable data on anaemia related maternal morbidity, proportional mortality rate due to anaemia in pregnancy could serve as a proxy indicator.
  There has been a steep decline in Maternal Mortality Ratio (MMR) from 398 (1997–98) to 130 (2014–16) per 100,000 live births in India [11]. Estimates suggest that 25% maternal deaths are due to haemorrhage, 18% due to anaemia [12, 13]. The estimated proportional contributors to mortality rates have remained unchanged since 1990.
  Therefore, a reduction in MMR with no concomitant rise in the proportion of anaemia related deaths probably suggests that mortality due to anaemia has reduced in India. However, it is very difficult to infer if the reduction is due to better management of anaemia during pregnancy because of better antenatal care (that raises Hb at the time of delivery) or better delivery care. Institutional deliveries in India have increased from 39% in 2005–06 to 79% in 2015–16, and further to 89% in 2019–20 [14]. The improved access may also translate into better availability of Emergency Obstetric services (EmOC) including trained birth attendants, blood transfusion during delivery resulting in better management and hence less mortality. The lack of change in anemia prevalence points against anemia reduction being the explanation for reduced MMR.
3. **Outcome measures of anaemia control programs**: One of the outcome measures of anaemia control programs is appropriate management that includes both prevention and treatment of anaemia in pregnancy. Apart from dietary counselling, the IFA tablets are distributed to pregnant women during the antenatal visits and those are captured in various surveys. Consumption of IFA tablets, though a better indicator, is difficult to capture due to its inherent challenges.
  Reliability of answers for a 5-year reference period on compliance of IFA supplementation is questioned due to recall bias, particularly those on numbers of tablets or bottles of syrups consumed during entire pregnancy. The protocol for data collection in the NFHS is not designed to capture this methodological-gap [15].
  A critical comparison between reduction of anaemia and distribution of IFA during pregnancy highlights this discrepancy. For instance, high reduction in the prevalence of anaemia was recorded between NFHS-3 and 4 in states such as Chhattisgarh (63–41%), Assam (72–44%), Haryana (71–51%), Odisha (68–47%) and Kerala (62–45%). However, the reported consumption of IFA supplementation among pregnant women (based on self reported data) was only 30.3% in Chhattisgarh, 32.0% in Assam, 32.5% in Haryana, 36.5% in Odisha and 67.1% in Kerala in NFHS-4 [6]. There is a need to undertake special surveys to report adherence to treatment.
4. **Differences in cut-offs to diagnose anaemia**: All NFHS surveys have taken a cut off value of 11 g/dl in pregnancy and 12 g/dl for non-pregnant women adjusted for smoking and altitude to define anaemia [8]. Evidence suggests that Hb content of the mother’s blood between 95 and 105 g/l in the third trimester correlates with the best clinical outcome [16, 17]. Considering the physiological aspects around pregnancy that results in haemodilution, the cut-offs in the second and third trimester of pregnancy should be 10.5 g/dl and 10 g/dl in the postpartum period [18]. NFHS surveys should be therefore interpreted with caution because of a uniform cut-off of 11 g/dl considered for analysis.
5. **Multifactorial etiology of anaemia**: Anaemia has a complex multifactorial etiology and so are the interventions for reducing it. Efforts cannot be limited to addressing iron deficiency anaemia alone [19]. Evidence shows that as the prevalence of anaemia and infection burden increases, the overall proportion of iron deficient anaemia reduces, even when ferritin concentrations are adjusted for inflammation. It is also found that the proportion of iron deficiency anemia among women of reproductive age is lower in countries with high prevalence of inflammation, which is often found as a component of infection [19].
  In Low and Middle Income Countries (LMICs), the most common cause of anaemia in pregnancy is iron deficiency [20, 21]. More than 60% of anaemia in India also is because of iron deficiency [3, 22]. However, other nutritional factors cannot be ignored. Low folate levels have been observed among adults in India especially among vegetarians [23]. In a study conducted on women in the reproductive age group in south India, folic acid deficiency was found in 56.8% and Vitamin B12 deficiency in 44.4% of women. Women with folic acid deficiency had roughly four times higher odds of having Vitamin B12 deficiency (OR, 3.86; 95% C.I. 2.71–5.49) [24]. Studies on pregnant mothers in India reveal that the prevalence of folate deficiency ranges between 20 and 30% [25, 26] and this is much higher than what has been reported from many other LMICs. In various studies, the prevalence of dimorphic anaemia varies from 13 to 26% in India [22, 27, 28]. Reports suggest that it could more than 50% also [29]. Vitamin A was reported in 3.8% (95% CI: 1.2–6.4%) of women in reproductive age group as reported from a study from southern India [30].
  Non nutritional causes also constitute a major burden that leads to anaemia. The prevalence of hemolytic anaemia in pregnancy in India varies from 4 to 11% [22, 27]. However, there are pockets in India such as northern and eastern belts and central India where the prevalence is likely to be high. Studies from India have reported varying prevalence of malaria in pregnancy (between 1 and 6%) [31, 32]. What is important to note is that afebrile malaria constituted around one fourth of malaria positive cases in pregnancy in another study [33]. Reports from scattered studies from developing countries on smaller samples suggest that the prevalence of worm infestation in pregnancy ranges from 11% to more than 25% [34–36]. Prevalence determined from small studies in India is reported to be around 12% [37]. Besides, improved sanitation is associated with a reduced risk of anemia by almost 12%. However, access to improved water is not associated with a reduction in anemia prevalence [38].

**Table 1:** Prevalence of moderate and severe anaemia in women of reproductive age (15–49 yrs)

States	NFHS-2 (1998–1999)		NFHS-3 (2005–2006)		NFHS-4 (2015–2016)	
	Severe Anaemia	Moderate Anaemia	Severe Anaemia	Moderate Anaemia	Severe Anaemia	Moderate Anaemia
Andhra Pradesh	2.4	14.9	3.3	20.6	1.9	18.5
Arunachal Pradesh	0.6	11.3	1.6	12.5	0.8	9.0
Assam	0.9	25.6	3.4	21.2	0.6	8.3
Bihar	1.5	19.0	1.0	15.9	0.7	13.9
Chhattisgarh			1.9	15.7	0.8	8.4
Goa	1.0	8.1	0.6	7.8	0.7	5.8
Gujarat	2.5	14.4	2.6	16.5	1.4	13.2
Haryana	1.6	14.5	1.7	16.7	1.4	18.4
Himachal Pradesh	0.7	8.4	1.2	10.5	0.7	13.0
Jharkhand			1.3	18.6	0.9	15.2
Karnataka	2.3	13.4	2.0	15.1	0.9	10.9
Kerala	0.5	2.7	0.5	6.5	0.3	4.4
Madhya Pradesh	1.0	15.6	1.0	14.1	1.1	12.2
Maharashtra	2.9	14.1	1.7	13.9	0.7	10.3
Manipur	0.8	6.3	0.5	5.1	0.3	4.0
Meghalaya	2.4	27.5	1.8	12.6	1.4	16.1
Mizoram	0.7	12.1	0.7	8.8	0.2	4.2
Nagaland	1.0	9.6			0.6	5.2
Odisha	1.6	16.4	1.5	14.9	0.7	9.8
Punjab	0.7	12.3	1.4	10.4	0.5	10.8
Rajasthan	2.1	14.1	2.5	15.4	1.0	11.2
Sikkim	2.4	21.4	1.7	16.2	0.6	7.2
Tamil Nadu	3.9	15.9	2.2	13.6	1.4	14.0
Telangana					2.5	17.3
Tripura					0.7	11.5
Uttarakhand			1.5	13.3	1.2	10.5
Uttar Pradesh	1.5	13.7	1.6	13.2	1.1	12.5
West Bengal	1.5	15.9	1.0	16.4	0.8	12.8
**India**	**1.9**	**14.8**	**1.8**	**15.0**	**1.0**	**12.4**

**Table 2:** Prevalence of anaemia according to severity in pregnant women (15–49 yrs)

States	NFHS-3 (2005–2006)			NFHS-4 (2015–2016)		
	Mild Anaemia	Moderate Anaemia	Severe Anaemia	Mild Anaemia	Moderate Anaemia	Severe Anaemia
Andhra Pradesh	28.8	26.2	3.4	26.0	25.0	1.9
Arunachal Pradesh	28.2	23.8	1.1	20.0	13.7	0.1
Assam	25.2	40.6	6.8	23.2	21.0	0.6
Bihar	28.8	29.9	1.7	28.4	28.9	1.1
Chhattisgarh	26.8	34.2	2.7	23.0	17.8	0.7
Delhi	15.3	13.4	1.2			
Goa	22.9	19.0	0.0	16.9	4.1	5.7
Gujarat	25.9	31.0	3.8	21.5	28.3	1.5
Haryana	26.0	43.1	1.9	23.6	29.4	2.0
Himachal Pradesh	27.5	12.8	0.0	23.5	26.1	0.8
Jharkhand	28.4	40.1	1.1	27.4	33.8	1.3
Karnataka	27.7	32.0	3.0	25.5	18.5	1.4
Kerala	19.7	15.5	0.0	16.6	6.0	0.0
Madhya Pradesh	23.6	33.1	2.0	25.5	27.5	1.7
Maharashtra	25.9	30.5	1.9	26.2	22.5	0.6
Manipur	19.9	16.7	0.6	16.0	8.8	0.4
Meghalaya	27.4	36.0	0.6	23.9	23.3	2.3
Mizoram	24.9	23.3	1.0	18.3	7.1	1.2
Nagaland	–	–	–	16.5	12.0	0.5
Odisha	37.1	29.6	2.0	24.0	22.7	0.8
Punjab	15.4	22.3	3.9	22.5	19.4	0.1
Rajasthan	23.7	35.3	3.1	20.3	24.7	1.6
Sikkim	22.6	36.9	2.6	15.3	8.2	0.0
Tamil Nadu	28.2	27.7	1.9	23.7	20.0	0.6
Telangana	–	–	–	21.4	26.7	1.6
Tripura	24.7	32.8	1.5	26.7	27.8	0.0
Uttarakhand	20.5	28.2	3.5	18.3	24.3	1.3
Uttar Pradesh	23.2	27.5	1.7	22.8	26.0	2.1
West Bengal	26.4	35.1	1.7	29.2	23.8	0.6
**India**	**25.8**	**30.6**	**2.2**	**24.5**	**24.6**	**1.3**

## Conclusion

Based on the above analysis it is very difficult to conclude that anaemia control program did not have any impact on the prevalence of anaemia in pregnancy in India. Serial cross-sectional surveys may not be the most ideal method to assess anaemia control programs. More research is needed to introduce better and accurate measurement methods for estimating Hb and its consistent use across large surveys. Advocacy efforts are warranted to revise the cut offs for more meaningful interpretation. Research is mandated to establish robust methods to ascertain the type of anaemia rather than focusing only on iron deficiency anaemia. Aggressive baseline mapping of anaemia and its determinants are required to assess the underlying causes. For better impact of anaemia control program on prevalence of anaemia in pregnancy, interventions should be geared up to address overall nutritional status of women of reproductive age group and adolescent girls. Research should focus on large scale implementation projects for enhancing the coverage of supplementation programs in the community.

## Data Availability

The data-sets analysed during the current study are available in public domain.

## Abbreviations

AMB: Anaemia Mukt Bharat
ANC: Antenatal care
DLHS: District Level Health Survey
EmOC: Emergency Obstetric services
Hb: Hemoglobin
IFA: Iron and folic acid
LMIC: Low and Middle Income Countries
MMR: Maternal Mortality Ratio
MoHFW: Ministry of Health and Family Welfare
NFHS: National Family Health Survey
NITI: National Institution for Transforming India
NIPI: National Iron Plus Initiative
NNACP: National Nutritional Anemia Control Programme
POSHAN: Prime Minister’s Overarching Scheme for Holistic Nutrition
